# Fully-automatic, patient-specific 3D aortic arch modeling for patient treatment with aortic arch anomalies

**DOI:** 10.1186/1532-429X-14-S1-O56

**Published:** 2012-02-01

**Authors:** Benedetta Leonardi, Dime Vitanovski, Allen Everett, Michael Suehling, Razvan Ionasec, Ludmilla Mantione, Giacomo Pongiglione

**Affiliations:** 1 Cardiology, Bambino Gesù Pediatric Hospital, Rome, Italy; 2 Corporate Technology, Siemens AG, CT T DE TC4, Erlangen, Germany; 3 Pediatric Cardiology, John Hopkins University, Baltimore, MD, USA

## Summary

Timing and type of aortic wall abnormalities (AWC) repair are still being debated. Automatically patient-specific 3D aortic arch geometrical model estimation from MRI images can provide a better knowledge of the geometry of the aortic arch anomaly and can be useful to evaluate preoperatively the best treatment. Therefore, we have developed a software to automatically compute a patient-specific 3D aortic arch geometrical model from CMR data and we have validated it.

## Background

Timing and type of surgical or transcatheter repair of aortic wall abnormalities (AWC) in patients with aortic coarctation (COA) and/or bicuspid aortic valve (BAV) are presently being debated, as associated morbidity and mortality can still occur. We have developed a system to automatically compute a patient-specific 3D aortic arch geometrical model from CMR data, which provides crucial information to understand the geometry of the pathophysiological abnormalities of the aortic arch and to evaluate preoperatively the best treatment.

## Aim

To validate the accuracy of the computed 3D geometrical model of the aortic arch by comparing manual measurements extracted directly from CMR images with the one automatically derived from the geometrical model.

## Methods

**Figure 1:**
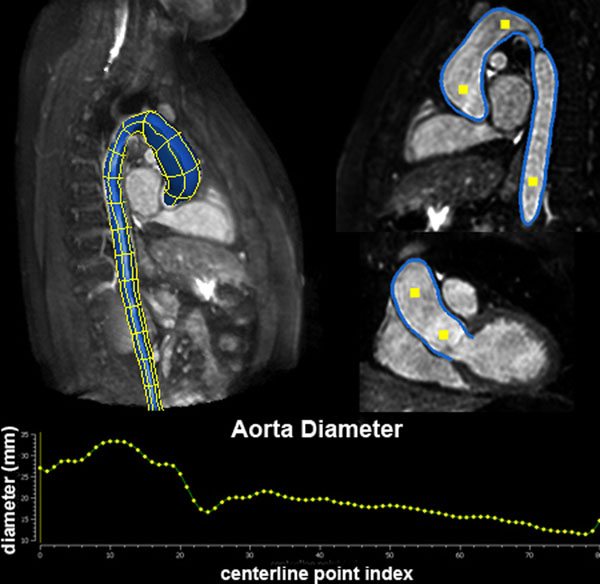
3D Aortic arch geometry automatically determined by our software from 3D SSFP sequence.

The system performance was evaluated on 32 patients with aortic arch anomalies (age: 5-36 years), 17 with COA and 15 with BAV and ascending aorta dilation. For reference, the aortic arch min and max diameters were measured manually from unenhanced, free - breathing, T2-prepared, segmented 3D SSFP sequence at aortic sinus (AS), sino-tubular junction (STJ), ascending aorta (AAO), transverse arch (TA), and descending aorta (DA). A computer-based, hierarchical model, which includes the aortic root, the ascending/descending aorta and the aortic arch, was estimated automatically from the CMR data using a novel machine learning algorithm (Figure 1). Diameter measurements at corresponding positions were then automatically derived from the computer-based model and compared with manual ones.

## Results

Statistical results significantly correlated (p < 0.001, r = 0.94) between min and max manual and automatic aortic measurements: AS (min p < 0.001 r = 0.85; max p < 0.001 r = 0.94), STJ (min p < 0.001 r = 0.88; max p < 0.001 r = 0.90), AAO (min p < 0.001 r = 0.94; max p < 0.001 r = 0.94), TA (min p < 0.001 r = 0.89; max p < 0.001 r = 0.93), DA (min p < 0.001 r = 0.90; max p < 0.001 r = 0.92).

Mean measurement error of 1.59±0.6 mm was achieved for the min diameter and 1.44±0.9 mm for the max diameter. The maximal error occurred at the minimum diameter of each segment with the STJ the greatest (min 2.07±2.53) and the DA the least (min 0.8±0.83).

Mean processing time for fully automatic aortic model estimation and measurement extraction was 1.5 s.

## Conclusions

Aortic parameters taken by our model are reliable, fully reproducible and faster as compared to manual methods. 2) The 3D aortic model is likely to improve therapeutic decision making in COA and/or BAV.

## Funding

No funding to disclose.

